# Pancreatic cancer associated with obesity and diabetes: an alternative approach for its targeting

**DOI:** 10.1186/s13046-018-0963-4

**Published:** 2018-12-19

**Authors:** Ramesh Pothuraju, Satyanarayana Rachagani, Wade M. Junker, Sanjib Chaudhary, Viswanathan Saraswathi, Sukhwinder Kaur, Surinder K. Batra

**Affiliations:** 1 Department of Biochemistry and Molecular Biology, University of Nebraska Medical Center, Omaha, NE USA; 2 Sanguine Diagnostics and Therapeutics, University of Nebraska Medical Center, Omaha, NE USA; 3 Department of Cellular & Integrative Physiology, University of Nebraska Medical Center, Omaha, NE USA; 4 Fred & Pamela Buffet Cancer Center, University of Nebraska Medical Center, Omaha, NE USA; 5 Eppley Institute for Research in Cancer and Allied Diseases, University of Nebraska Medical Center, Omaha, NE USA

**Keywords:** Pancreatic cancer, Obesity, Insulin resistance, Diabetes, Adiponectin, Leptin, Gut microbiota, Inflammation

## Abstract

**Background:**

Pancreatic cancer (PC) is among foremost causes of cancer related deaths worldwide due to generic symptoms, lack of effective screening strategies and resistance to chemo- and radiotherapies. The risk factors associated with PC include several metabolic disorders such as obesity, insulin resistance and type 2 diabetes mellitus (T2DM). Studies have shown that obesity and T2DM are associated with PC pathogenesis; however, their role in PC initiation and development remains obscure.

**Main body:**

Several biochemical and physiological factors associated with obesity and/or T2DM including adipokines, inflammatory mediators, and altered microbiome are involved in PC progression and metastasis albeit by different molecular mechanisms. Deep understanding of these factors and causal relationship between factors and altered signaling pathways will facilitate deconvolution of disease complexity as well as lead to development of novel therapies. In the present review, we focuses on the interplay between adipocytokines, gut microbiota, adrenomedullin, hyaluronan, vanin and matrix metalloproteinase affected by metabolic alteration and pancreatic tumor progression.

**Conclusions:**

Metabolic diseases, such as obesity and T2DM, contribute PC development through altered metabolic pathways. Delineating key players in oncogenic development in pancreas due to metabolic disorder could be a beneficial strategy to combat cancers associated with metabolic diseases in particular, PC.

## Background

The pancreas contains exocrine and endocrine cells. The endocrine cells secrete insulin, glucagon, and somatostatin, whereas exocrine cells are involved in the secretion of digestive enzymes. Pancreatic cancer (PC) is lethal malignancy and approximately, 95% of PC has an exocrine cell origin. It is very difficult to diagnose at an early stage due to the lack of symptoms and deep retroperitoneal of pancreas. This PC type is commonly  known as pancreatic ductal adenocarcinoma (PDAC), with a 5-year survival rate of ~7.2% in the United States (US) [1]. PC has become the third leading cause of cancer-related deaths with an estimated new cases of 55,440 and deaths of 44,330 in 2018 [2]. The lifetime risk of developing PC in any one person is 1.6% and it is expected to surpass colon cancer in mortality by year 2030 [3]. PC is frequently diagnosed at an advanced stage, when the cancer has metastasized to distant organs like the liver, lung, lymph node and peritoneal cavity [4]. Unfortunately by the clinical presentation, 85% of the tumors are unresectable [5, 6] which translates to poor prognosis and high mortality in the absence of effective chemo- and radiotherapies. Risk factors for PDAC include age (high percentage in elderly), sex (high incidence in men), gene mutations, cigarette smoking (nearly one quarter of all PC cases), obesity, chronic pancreatitis, and diabetes [7, 8].

In PC, pancreatic stellate cells form a dense stromal tissue, which is referred to as a desmoplastic reaction. Stellate cells are responsible for limiting vascularization, which leads to hypoxia, tumor progression, invasion, and metastasis [9–13]. In PC, a compendium of mutations occur in various oncogenes like Kirsten rat sarcoma viral oncogene homolog (*KRAS*) and tumor suppressor genes (*INK4A/p16*, *Tp53* and *SMAD4*) [14]. Mutations in the *KRAS* oncogene, observed in more than 90% of PC tumors, leads to constitutively active Ras protein that results in uncontrolled cell proliferation. Further, inactivating mutations in *INK4A/p16* and *Tp53* results in the loss of cell cycle and apoptotic regulation [4]. Differential expression of epidermal growth factor receptor (*EGFR*), mucins (*MUC1*, *MUC6* and *MUC5AC*) and matrix metalloproteinases (MMPs) occurs during precursor development [15]. Mutations in *INK4A/p16* (90%) appear in PanIN-2, whereas *Tp53* (85%) and *SMAD4* (55%) mutations are found in PanIN-3. Since PanINs represent precancerous ductal lesions, these mutations are considered early molecular biomarkers for PC [15]*.* A combination of biomarkers (EGFR, ERK, SIAH, Ki67 and HIF-α) can predict survival rates for patients with resectable PC. In fact, a combination of these biomarkers is more strongly associated with pathological features including tumor size, tumor grade, margin and lymph node status compared to a single marker [7, 16, 17]. In a multicenter study, to differentiate PC from chronic pancreatitis and their benign controls, mucin (MUC5AC) alone or in combination with CA19-9 could be a potential diagnostic/prognostic biomarker [18].

Due to generic symptoms (weight loss, fatigue, jaundice, abdominal pain and nausea) common across  multiple other pathologies , early identification of PC is difficult [19, 20]. Recent studies suggest that PC develops from a precursor lesion of <5 mm in diameter and may take an average of 20 years to metastasize [20]. Therefore, it provides a window of opportunity to diagnose and treat PC if it is detected at an early stage [21]. To date, efforts are being made in multiple directions to develop early diagnostic test for PC including histopathological tests on fine needle aspirates, serological tests, imaging (computed tomography/magnetic resonance imaging), and analysis of genetic mutation markers [21–23]. Regarding PC treatment, gemcitabine (a nucleotide analogue) is the preferred first-line option but survival is often less than ~5 months. Combination therapy with gemcitabine and erlotinib (an inhibitor of EGFR) increased the 1-year survival rate to 23% as compared to 17% in the gemcitabine plus placebo group in a randomized phase III clinical trial [24]. Other drugs such as folfirinox/nab-paclitaxel with gemcitabine also increase survival [25–27]. In a clinical trial, metastatic PC patients were treated by administration of folfirinox (5-fluorouracil with leucovorin, irinotecan, and oxaliplatin) had shown greater efficacy for metastatic cancer; however, few limitations were observed due to its cytotoxicity [28]. However, in a systematic study, over 30 years (from 1986 to 2016) weighted median overall survival was improved with folfirinox alone [3]. In addition to the above chemotherapeutic agents, different treatment options for PC patients includes Capecitabine and 5-fluorouracil (5-FU) along with platinum-based or other cancer drugs (leucovorin, exatecan, and irinotecan) [27]. Therefore novel treatment strategies are needed to improve the overall survival in PC patients.

### Obesity, insulin resistance and diabetes

Obesity has become a serious threat worldwide and is considered an epidemic. It occurs due to changes in lifestyle (physical inactivity, intake of high fat/caloric diet, high sugar diet) and is also associated with lifestyle including cigarette smoking and alcohol consumption. Additionally, genetic factors such as mutation in the leptin pathway leads to monogenic obesity while chromosomal abnormalities results in syndromic obesity [29]. In the body, adipose tissue (AT) plays an important role in the storage of triglycerides (TG), which come from the diet. It is classified as brown and white AT, where brown AT (BAT) is predominantly located in the cervical area and utilizes TG to generate heat (a process called as thermogenesis). Disappearance of BAT has been observed during the aging process and recently it has gained significant attention. White AT is present in the subcutaneous layer, omentum and retroperitoneal cavity, where it stores excess fat. According to the lipid burden hypothesis, AT stores sufficient lipids in the form of droplets. Excess storage of lipids leads to hypertrophy (increase in cell size) and hyperplasia (increase in cell number) [30]. Moreover, in obesity, heavy traffic of lipids inside the body leads to release of excess TG in the form of free fatty acids (FFAs) into the circulation. Further, these FFAs accumulate in non-adipose tissues such as the pancreas, muscle, liver, heart and kidney, resulting in insulin resistance and diabetes [31].

**Fig. 1:**
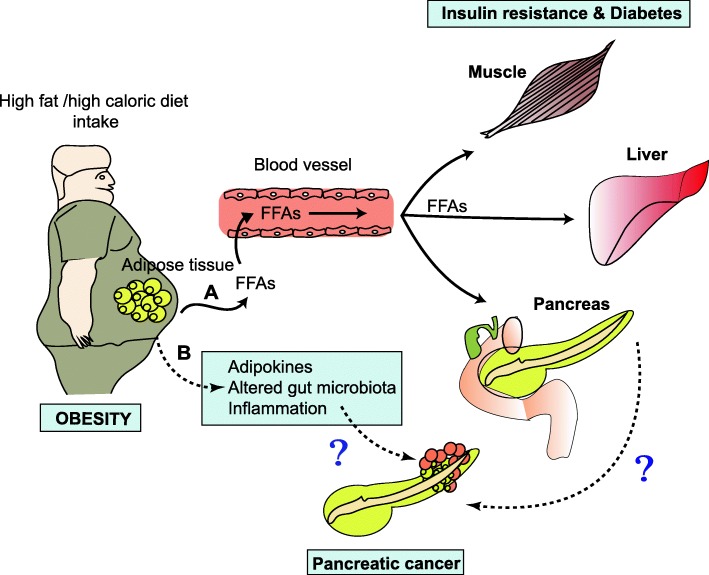
A schematic representation of obesity- and diabetes-associated pancreatic cancer. High fat/caloric intake results in accumulation of excess fat, which further leads to development of obesity. **a**. In obesity, adipose tissue releases free fatty acids (FFAs), which enter circulation and accumulate in the non-adipose tissues such as muscle, liver and pancreas that leads to insulin resistance and diabetes. **b**. Along with FFAs, adipokines, altered gut microbiota and inflammatory markers contribute to pancreatic cancer development through unknown mechanisms.

Obesity is a multifactorial disease associated with several metabolic disorders including insulin resistance, glucose intolerance, dyslipidemia, and elevated blood pressure. All these disorders are collectively called metabolic X syndrome [32]. Further, obesity is a strong risk factor for type 2 diabetes mellitus (T2DM), cardiovascular diseases and even many types of cancers such as pancreatic, hematological, prostate and breast cancers [33]. Recent studies have revealed that obesity and PC are strongly associated. For instance, a body mass index greater than 35 is one of the risk factors for PC in both men and women [33, 34]. Moreover, studies have suggested that both obese mice and patients develop PC lesions following an increase in fat mass [35, 36] and show infiltration of fat cells in the pancreas as a consequence of PC development [37, 38]. Insulin resistance is a hallmark of T2DM, in which insulin fails to trigger adequate glucose uptake, leading to accumulation of circulatory glucose as well as increased insulin levels. These increased insulin levels in T2DM patients may be associated with PC growth by binding to its receptors located on the pancreas. For example, we still don’t know if the insulin resistance that characterizes T2DM promotes PC or if the reverse is true (Fig. 1). In the present review, we have attempted to compress all the available literature on obesity-and diabetes-associated molecules involved in PC development. Several molecules have been characterized in obesity-associated PC, whereas less is known about factors unique to diabetes-associated PC. These molecules are expected to be the focus for future investigations of the molecular oncology of cancer.

### Obesity associated pancreatic ductal adenocarcinoma

Obesity is associated with pancreatic and other types of cancers [39–41]. Individuals with abdominal adiposity have a 50% increased risk of PC development compared to lean individuals [42]. In the U.S., about 70% of the adult population is overweight and has a two-fold increased risk of PC incidence and mortality [39, 42]. However, link between obesity and PC is still not fully understood [43]. The current theory is that excess TG in obesity leads to an increase in size and number of adipocytes, which results in devascularization, hypoxia, and ultimately macrophage infiltration. In this condition, adipocytokines including adiponectin, leptin, tumor necrosis factor-alpha (TNF-α), interleukins, and monocyte chemoattractant proteins are secreted locally leading to inflammation. Evidence suggests that increased levels of adipocytokines, altered gut microbiota, and inflammation are involved in PC progression [39, 44]; thus, this review focuses on the possible oncogenic roles of these factors in PC.

#### Adipocytokines

Besides storing excess energy as TG, AT secretes several factors regulating energy metabolism in various organs. These adipokines including adiponectin, leptin, resistin, and ghrelin play an important role in glucose and lipid metabolism. Among them, adiponectin and leptin are the most important and are therefore the focus here in discussing obesity-associated PC.

#### Adiponectin

**Fig. 2:**
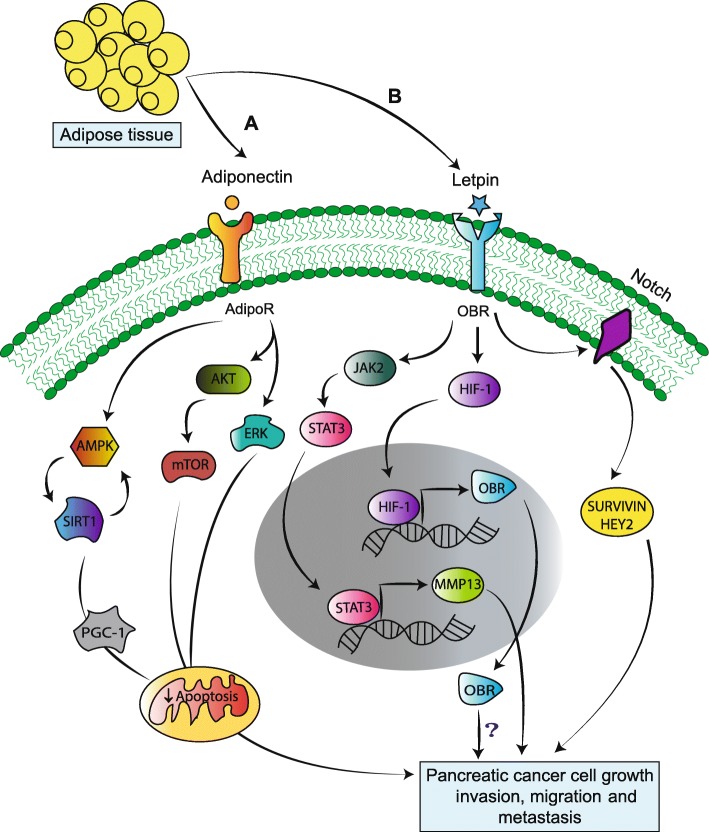
Adipocytokines mediate pancreatic cancer tumorigenesis by different signaling mechanisms. **a**. Adiponectin secreted from adipose tissue binds to its receptor (AdipoR) to activate AKT, MAPK and AMPK pathways, which block the apoptosis of pancreatic cancer cells. **b**. Similarly, leptin binding to its receptor (OBR) results in activation of the JAK2/STAT3 pathway, which leads to matrix metalloproteinase-13 activation and eventual pancreatic cancer metastasis. In addition, OBR also regulates its own expression through hypoxia inducible factor-1, resulting in cancer cell survival via an unknown mechanism. Moreover, leptin also triggers Notch receptor signaling, which results in activation of its downstream molecules (survivin and Hey2), thereby increasing cancer cell proliferation.

Adiponectin is also referred to as AdipoQ, which acts on several tissues to control energy homeostasis and insulin sensitivity [45, 46]. It regulates carbohydrate as well as lipid metabolism through the adenosine monophosphate-activated protein kinase (AMPK) pathway. The expression of circulatory AdipoQ is decreased in obesity and diabetes. However, the role of circulating AdipoQ in PC remains debatable regarding its impact on pancreatic tumor progression. Adiponectin serve as negative regulator that mediate its function by acting on its two receptors i.e. AdipoR1 and AdipoR2. Mechanistically, AdipoQ increases insulin synthesis and secretion by preventing apoptosis of pancreatic β-cells through activation of ERK and AKT pathways [47] (Fig. 2). Huang et al. demonstrated that subcutaneous implant of mouse pancreatic cell lines (H7 and Panc02) in AdipoQ knockout (APNKO) mice has reduced tumor weight and size as well as increased apoptosis by up-regulating cleaved caspase-3 as compared to wild type (WT) littermates. In addition, knockdown of AdipoR1, the major receptor of AdipoQ in these mouse cell lines (H7 and Panc02) followed by subcutaneous injection reduced tumor weight, size, and expression of Ki-67 (proliferation marker). Further, AdipoQ was observed to decreases apoptosis and increases PC cell proliferation and migration by activating the AMPK-Sirt1-PGC1α pathway [48] (Fig. 2). Similarly, in a case-control study, Dalamaga et al. studied the blood levels of AdipoQ in PC and control cases both before and after controlling for age, gender, BMI, smoking status, alcohol consumption, history of diabetes, and family history of PC. Higher AdipoQ levels were associated with PC. At tissue level, utilizing 16 tumor tissues, the authors observed positive or strong positive expression of AdipoR1 in 87.5% of cases while positive or strong positive expression of AdipoR2 was observed in >97% cases. Based on this, the investigators suggested to investigate the role of AdipoQ as a marker for early detection of PC. Further, Kadri et al. observed no correlation between adiponectin levels and PC [49]. Similarly, Pezzilli et al. did not observe any significant correlation among adiponectin levels and PC at serum level [50]. However, retrospective and prospective studies indicate that early detection of low circulatory AdipoQ levels may or may not be associated with the development of PC, because single nucleotide polymorphisms of the AdipoQ gene are common [51–54] and the presence of these SNPs in AdipoQ, but not its receptors, are associated with altered serum adiponectin levels [55].

Inhibitory role of AdipoQ in halting tumor progression has also been observed [49]. In this regard some clinical studies suggest that circulating AdipoQ inhibit tumor cell proliferation by decreasing AKT and beta catenin levels across multiple malignancies (breast, colon and prostate) [56, 57]. In the case of PC, the molecular mechanism by which up-regulated AdipoQ levels inhibit cancer progression is still unclear; possibilities include 1) increasing insulin sensitivity via phosphorylation of insulin receptors, which down-regulates insulin/IGF-1 signaling, 2) down-regulating the expression of inflammatory cytokines that inhibit NF-κB activation, 3) directly activating the AMPK pathway to activate the p53 tumor suppressor gene, and 4) promoting cancer cell apoptosis via peroxisome proliferator-activated receptor gamma (PPARγ) activation and inhibiting angiogenesis [58, 59]. One study fed genetically engineered PC mice (Kras^G12D^/Pdx-1-Cre) with a calorie-restricted diet and observed delays in formation of pancreatic intraepithelial neoplasms (PanIN) [60, 61]. Delayed progression of PanIN to PDAC was accompanied by increased AdipoQ and Sirt1 levels as well as decreased mTOR and IGF-1 expression [61]. In another study, Kato et al. incubated recombinant AdipoQ with the Pan02 murine cell line and noted decreased cell proliferation and increased apoptosis at 5 and 10 μg/ml, respectively. Further, orthotopic implantation of Pan02 cell line showed a significant increase in tumor volume by higher vascularization (more microvessel density) and decreased apoptosis in AdipoQ knockout mice as compared to WT animal [58, 62]. Overall, the findings from this study suggested AdipoQ to be a tumor suppressive role in PC by directly inhibiting proliferation and inducing apoptosis [62]. Interestingly, a recent study by Messaggio and co-workers showed that the decreased expression of AdipoQ receptors in pancreatic tumor tissues as compared to adjacent normal tissue. To elucidate the role of AdipoQ, its agonist AdipoRon was applied to both mouse and human cell lines and was found to inhibit PC tumor growth and proliferation by down-regulating leptin-induced STAT3 signaling. These results suggest AdipoRon could be a potential therapeutic agent for PC [63].

#### Leptin

Leptin was the first adipokine identified in AT in 1993; it controls food intake and energy expenditure *via* a feedback mechanism in the brain [64]. After secretion from AT, leptin enters into circulation and reaches a level depending upon the AT size [65]. Under normal physiological conditions, leptin decreases appetite and increases fatty acid oxidation through its receptor (OBR or LEPR). However, in obesity and diabetes, elevated circulatory levels of leptin do not drive the same appetite feedback responses [66]. Like AdipoQ, leptin has a role in PC pathogenesis. In PC tumor cells, leptin binds to both full-length receptor (OBR1) as well as the short form (OBRs) to mediate downstream signaling [67]. Leptin receptor (OBR) and hypoxia inducible factor-1 (HIF-1) are predominantly co-expressed in PC cell lines and tissues during hypoxic conditions. HIF-1 binds to the hypoxia-responsive element (HRE) in the OBR promoter, regulating OBR transcription. Co-expression of OBR and HIF-1 in PC tissues was associated with poor prognosis, decreased overall survival and increased metastasis to distant organs in PC patients (Fig. 2). Silencing of HIF-1 inhibited leptin receptor expression in PC cells, suggesting that a positive feedback loop between HIF-1 and leptin/OBR mediates PC progression [67]. In another *in vitro* study, recombinant human leptin promoted PC cell migration and invasion but had no effect on proliferation [68]. The migration of PC cells occurred via the janus kinase 2 and signal transducer and activator of transcription 3 (JAK2/STAT3) pathway, which targets its downstream effector matrix metalloproteinase 13 (MMP13). The *in vivo* impact of leptin-over expressing PC cells was tested by orthotopic implantation into athymic nude mice, which led to greater tumor growth and lymph node metastasis. Over expression of leptin in PC cells and mouse tumors resulted in up-regulation of MMP13 levels, suggesting that leptin/MMP13 signaling is important for metastasis. In addition, MMP13 levels correlated with OBR expression in lymph node metastatic human PC tissues. The authors concluded that PC cell migration, invasion and metastasis occur via the JAK2/STAT3/MMP13 pathway [68] (Fig. 2).

A high fat/caloric diet leads to obesity, insulin resistance and increased leptin levels, all of which contribute to pancreatic adiposity. The accumulation of lipid molecules into the pancreas leads to activation and deposition of inflammatory cytokines (e.g., interleukin-6), which potentiate PC cell growth, migration and invasion [69]. Leptin activates Notch signaling and its receptors, leading to activation of its downstream molecules (survivin and Hey2) required for PC proliferation (Fig. 2). Notch signaling also up-regulates stem cell markers (CD44, CD24 and ESA) in PC cells. Inhibition of leptin (by IONP-LPrA2) after subcutaneous implantation of PC cells delayed tumor onset and decreased tumor size as well as cancer stem cell markers [70]. In another study by the same group reported that BxPC-3 and MiaPaCa-2 PC cells were treated in the presence of 5-FU, leptin, notch inhibitor (DAPT) and leptin inhibitor (IONP-LPrA2). They observed that decreased 5-FU cytotoxicity (by decreasing pro-apoptotic markers), increased cell proliferation and anti-apoptotic factors was due to leptin treatment. Moreover, IONP-LPrA2 reduced PC tumorspheres (treated with 5-FU) *via* notch signaling and suggesting that leptin might be involved in reducing the cytotoxic effect of chemotherapeutic drug and facilitating chemoresistance [71]. The leptin-notch signaling axis targeting has been projected as potential mediator for benefitting PC patients with obesity. Overall, the effect of AdipoQ and leptin in the progression of PC is still under investigation in obese people and further studies are warranted before targeting these adipokines in PC therapy.

#### Gut microbiota and inflammation

**Fig. 3:**
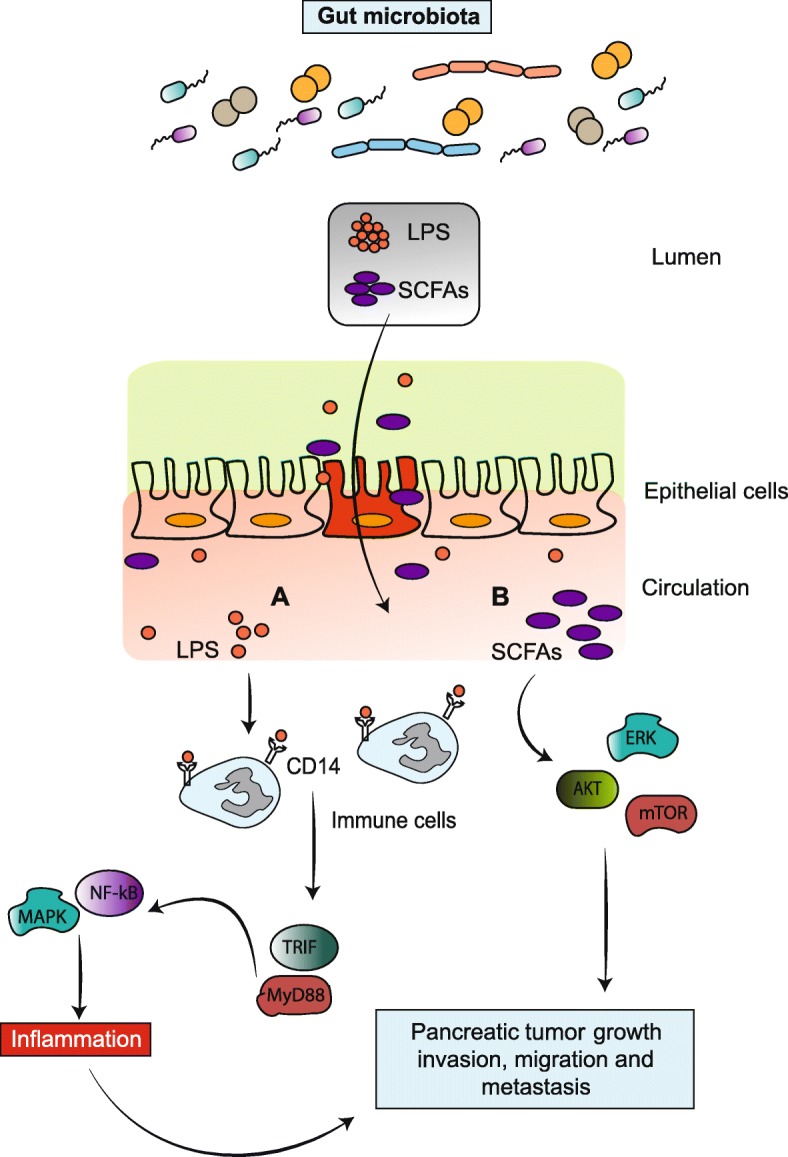
Altered gut microbiota is responsible for pancreatic cancer progression. **a**. High-fat diet intake alters the gut microbiota composition. Altered gut microbiota secrete lipopolysaccharides (LPS), which enters circulation by damaging intestinal tight junction proteins. Circulatory LPS then binds to the toll like receptor on immune cells to recruit MyD88 or TRIF adaptor molecules. These molecules further activate MAPK and NF-κB pathways to activate  several inflammatory cytokines, leading to cancer cell proliferation. **b**. Short-chain fatty acids (SCFAs) are released from resistant starch by the gut microbiota that enters the circulation. Afterward, SCFAs bind to G-protein-coupled receptors to activate the MAPK signaling pathway, triggering cancer cell proliferation.

The gut microbiome (hidden organ) comprises at least 10^14^ microorganisms belonging mostly to the phyla *Firmicutes* and *Bacteroidetes*, which play an important role in obesity and other metabolic disorders [72]. Recent evidences suggest that diet, environmental factors and microbial components can contribute to the development of cancer in liver and pancreas through a gut-liver/pancreas axis [73]. As shown in Fig. 3, a high-fat diet can alter the gut microbiome and trigger an inflammatory cascade. Gram-negative bacteria secrete lipopolysaccharide (LPS), which induces low-grade inflammation through its binding to toll-like receptors (TLRs) and CD14 co-receptors present on monocytes, macrophages and neutrophils [74, 75]. Furthermore, altered gut microbiota may lead to decreased intestinal tight junction proteins (ZO-1 and occludin), which allows LPS entry into circulation [76]. Binding of LPS to its up-regulated receptors (CD14 or TLRs) on immune cells induces PC cell proliferation [77, 78]. Additionally, these immune cells also play a role in cancer cell invasion, angiogenesis and metastasis [79–81] by recruiting myeloid differentiation primary response gene 88 (MyD88) or TIR-domain-containing adapter-inducing interferon-β (TRIF) adaptor molecules. Activation of these molecules leads to inflammation by up-regulating p44/42 mitogen-activated protein kinase/extracellular signal-regulated kinase (MAPK) and NF-κB pathways (Fig. 3). Therefore, an altered gut microbiota may promote cancer by driving inflammatory responses [82]. In support of this, germ-free (absent microflora) mice are less prone to carcinogenesis probably due to a decrease in tumor-associated inflammation [83, 84]. Similar results were observed when WT mice were treated with broad-spectrum antibiotics to inhibit the microbiota [85]. As final evidence, antigenic peptide secreted from *Helicobacter pylori* (which causes gastric ulcers) has been associated with PC pathogenesis [86]. *H. pylori* components translocate into the pancreas from the gut and activate NF-κB, thereby increasing the expression of pro-inflammatory cytokines involved in PC initiation and progression [87]. A recent study by Sethi et al. demonstrated that the gut microbiome modulation may have impact on tumor growth in a mouse model. Initially, the authors orally administered a cocktail of broad-spectrum antibiotics to C57BL/6J mice for 15 days. Then at 15 days, a pancreatic cell line derived from *Kras*^*G12D/+*^*; Trp53*^*R172H/+*^*; Pdx1*^*cre*^ (KPC) mice was injected subcutaneously or intrasplenically (to induce liver metastasis). Results of this study showed that absence of gut microbiota led to a significant decrease in subcutaneous tumors, and decreased degree of liver metastasis. Besides, an absence of gut microbiota shows a significant increase in anti-tumor mature T cells [Th1 (IFN gamma^+^CD4^+^CD3^+^) and Tc1 cells (IFN gamma^+^CD8^+^CD3^+^)] in the tumor microenvironment with an unknown mechanism. Finally, the relative abundance of *Bacteroidetes* and *Firmicutes* phyla decreased in fecal samples upon antibiotic administration in KPC mice. The authors concluded that modulation of gut microbiota on tumor progression could be a novel immunotherapeutic strategy [88].

In general, pancreatic tumors depend on carbohydrate metabolism for their survival, growth and resistance to chemotherapy. Dietary carbohydrates are usually fully metabolized in the small intestine, with the exception of resistant starch. Gut microbiota further process the starch in the large intestine through fermentation, and as a result, short-chain fatty acids (acetate, butyrate and propionate) are released. Resistant starch by avoiding degradation in small intestine imparts several health benefits via decreasing circulatory glucose levels, body weight, and inflammation without causing any side effects [89]. Interestingly, media engineered to mimic resistant starch (low glucose concentration) decreased PC cell proliferation compared with control media. The decrease in cell proliferation is due to down-regulation of ERK and mTOR signaling (Fig. 3). Similarly, mice bearing sub-cutaneous PC tumors fed a resistant starch diet showed lower tumor weight than controls on a normal diet. Additionally, resistant starch also inhibits the growth of inflammation-causing organisms including *Bacteroides acidifaciens*, *Ruminococcus gnavus*, *Clostridium cocleatum* and *Escherichia coli* in mice by modulating gut microbiota [90].

Early metastasis (primarily at lymph nodes and liver) and chemoresistance are responsible for PC aggressiveness. However, treatment with gemcitabine, first line therapy for metastatic PC, results in altered gut microbiota, which affects PC growth. Administration of gemcitabine in nude mice bearing subcutaneous PC cell line tumors leads to increased growth of *Proteobacteria* and *Akkermansia muciniphila,* which potentiate inflammation and/or mucin degradation. The imbalance of gut microbiome due to gemcitabine treatment also disrupts the intestinal integrity; this, in turn, favors the entry of microorganisms or their components into the circulation to reach distant organs. In the pancreas, the microbe-associated molecular patterns (such as LPS and endotoxins) on the microbial surfaces bind to TLRs, activating inflammation through NF-kB signaling. In addition, gemcitabine-treated mice have greater LPS-induced inflammation and lower levels of inosine (a naturally occurring metabolite of adenosine), which has anti-inflammatory and immunosuppressive effects [91]. Furthermore, fecal microbiota obtained from KPC mice was recolonized into antibiotic treated WT mice which shows higher bacterial population access into the pancreas. Ablation of gut microbiota in *Ptfla*^Cre^; LSL*-Kras*^G12D^ (KC) mice by oral antibiotics were recolonized with feces derived from WT or KPC mice and pancreatic tumor growth acceleration was observed in KPC derived feces only. Similarly, recolonization of feces (from KPC animal bearing pancreatic tumors) in germ free (GF)-KC mice shows increased pancreatic tumor growth as compared to GF-WT mice. This tumor acceleration might be associated with a decrease in activated T-cell infiltration in GF condition. They hypothesized that antibiotic treatment results in an increased intratumoral CD8:CD4 T-cell ratio which activates immunogenicity in PC. Future studies are warranted to identify microbial signatures that influence growth of PC tumors [92]. Taken together, a better understanding of the role of gut microbiota in PC tumor progression could open up new avenues in PC therapy development.

In obesity, pro-inflammatory cytokines are released from AT macrophages and infiltrate into AT; however the exact mechanism for these events is not known. In obese rats and humans, elevated inflammatory cytokine TNF-α activates other cytokines, in particular, IL-6, promoting angiogenesis and metastasis [93–95]. Therefore, the possible common mechanism by which obesity induces inflammation in several cancers (pancreatic, lymphoma and glioblastoma) might be through TNF-α-induced NF-κB signaling [96–98]. In addition, TNF-α secreted from cancer cells triggers cancer associated fibroblasts to stimulate macrophage infiltration [99, 100]. This infiltration occurs in several cancers through TNF-α-induced IL-6 to up-regulate STAT3 signaling [101]. Mice with PC tumors and diet-induced or genetic obesity expressed significantly higher STAT3 in the PC tumors. The up-regulation of STAT3 can drive PC progression through the activation of anti-apoptotic and proliferative proteins (Bcl-X_L_, Mcl-1, Survivin, c-Myc and cyclin D1) as well as matrix metalloproteinases [102–104]. Currently, studies are focused on the role of AT-derived inflammatory cytokines in modulating signaling pathways that can indirectly influence the progression of PC.

#### Glucose metabolic enzymes

Despite the harsh hypoxic environment, PC survives in part due to expression of HIF1-α, which prevents apoptosis and increases the synthesis of glycolytic enzymes and transporter proteins [105]. According to the Warburg effect, the cancer cell depends on glycolysis to produce energy instead of aerobic respiration [106–108]. The most important rate-limiting glycolytic enzymes are pyruvate kinase (PKM2), which catalyzes the conversion of phosphoenol pyruvate to pyruvate, and lactate dehydrogenase (LDHA), which then catalyzes conversion of pyruvate to lactate. The glycolytic pathway releases high-energy phosphates in the form of nicotinamide adenine dinucleotide, which enters the mitochondria for energy synthesis. LDHA is overexpressed throughout carcinogenesis, while PKM2 expression increases during the transition of cystic lesions to cancer. A possible explanation is that cystic lesions require high levels of LDHA, which induces PKM2 splicing in a later stage of tumor proliferation [109]. Furthermore, activation of EGFR initiates translocation of PKM2 to the nucleus where it binds to β-catenin, resulting in up-regulation of cyclin D1, Stat3, Oct4 and HIF, which induce cell proliferation [110, 111]. Therefore, both glycolytic enzymes (PKM2 and LDHA) are possible targets for PC treatment in preclinical studies.

#### Hepatocyte growth factor

In addition to adipokines, pre-adipocytes as well as mature AT secrete cytokines and growth factors that have a role in tumor growth. In pancreatic tumor progression, cross-talk between PSC and PC is mediated through several growth factors including platelet-derived growth factor, transforming growth factor, vascular endothelial growth factor and hepatocyte growth factor (HGF) [112, 113]. HGF has received much attention due to its mitogenic signal and its angiogenic effects on AT [114, 115]. In the case of obesity, HGF is released from the AT and the resulting circulatory levels contribute to pancreatic cell proliferation [116]. Exogenous supplementation of HGF induces proliferation in a murine pancreatic cell line (Pan02) through its receptor c-MET, whereas in the absence of c-MET, HGF had no direct effects in a murine pancreatic cell line and indirectly inhibited apoptotic cell death [117]. HGF inhibition by means of neutralizing antibody (AMG102) inhibited tumor growth and metastasis as compared to gemcitabine treatment [118]. Over expression of c-Met renders PC cells resistant to gemcitabine and radiation [44, 119] through an unknown mechanism. As one possibility, Cui and co-workers demonstrated that the Forkhead box M1 (FOXM1) transcription factor regulates c-MET expression via ERK, AKT and STAT3 pathways, creating a positive feedback loop that promotes tumor growth. Further, inhibition of c-MET, FOXM1, ERK, AKT and STAT3 signaling pathways with their respective inhibitors abolished the c-MET positive loop [120]. Therefore, the HGF/c-MET feedback loop regulates tumor proliferation, invasion and migration [121] and may be a novel target for growth factor-induced tumor growth.

#### Hyaluronan

In obesity, TG accumulates in the pancreas along with other organs and results in inflammation, higher expression of cytokines and remodeling of extracellular matrix (ECM). Hyaluronic acid or hyaluronan (HA) is a glycosaminoglycan and ubiquitous component of ECM which increases interstitial fluid pressure (IFP) and also reduces entry of chemotherapeutic drugs in PC tumors [122]. In tumor progression, the cross-talk between cancer cells and ECM is very important. Normally, HA synthesized by hyaluronan synthase (HAS) and secreted into the ECM under controlled conditions. However, increased expression of HA was observed in insulin-resistant mice aorta [123] and in the pancreas of diabetic mice [124]. In addition, expression of HA in the ECM is associated with diet-induced insulin resistance and was reversed upon treatment with the drug pegylated recombinant human hyaluronidase (PEGPH20), which improves insulin sensitivity in muscle tissue [125].

The PC stroma cells and the ECM express abundant HA to maintain a supportive tumor microenvironment [126]. Binding of HA to its receptors [cluster of differentiation-44 (CD44) or receptor for HA-mediated motility (RHAMM)], activates Ras and PI3K signaling, leading to increased cell proliferation, migration, and metastasis. Further, the activated PI3K pathway in cancer cells also increases drug resistance via activation of a multi-drug receptor [127–129]. The HA receptor CD44/RHAMM mediates cell-cell/matrix interactions and up-regulation of HA (around 12-fold increase) is observed in PC [130–133]. PC cells increase expression of HA via epigenetic regulation (decreased DNA methylation) and concomitant up-regulation of its enzyme HAS [134]. HA exists in low and high molecular weight forms. *In vitro* treatment with low molecular weight HA (25-75 kDa) increased PC cell motility compared to treatment with high molecular weight HA (400-600 kDa) [135, 136]. In conclusion, inhibition of HA synthesis may be a possible therapeutic strategy against PC and obesity-associated PC. Recently, PEGPH20 has gained interest to target HA for improving intratumoral microenvironment in PC. The different concentrations of HA along with mouse PC cells were implanted in immunodeficient mice that showed high IFP which reduce delivery of chemotherapeutic drugs. So targeting HA, a single high dose of PEGPH20 had a significant reduction on IFP in KPC mice. Further, a combination of PEGPH20 and gemcitabine showed decrease in cell proliferation and increased apoptosis in KPC mice [137]. In a randomized phase II clinical study, metastatic PC patients (231 were selected from a total of 279 patients) were treated with nab-paclitaxel/gemcitabine (AG) or PEGPH20 + nab-paclitaxel/gemcitabine (PAG). Patients (n=84) who had HA-high tumors showed improvement in the progression-free survival, overall survival and reduction in the thromboembolic (TE) incidence by PAG alone. Furthermore, PAG treatment was accompanied by more muscle spasm, neutropenia, myalgia and TE as compared to AG. Overall, srudy finding suggested that tumor HA could be a promising therapeutic target for PC patients with high HA [138].

### Diabetes mellitus associated pancreatic ductal adenocarcinoma

Obesity is associated with insulin resistance and T2DM, which in turn is a potential risk factor for PC. In a post-prandial state, insulin maintains the levels of circulating glucose and FFAs. Insulin resistance is a condition in which the adipose and muscle tissues and to a lesser extent the pancreas, brain, liver and kidney are unable to respond to insulin. Insulin resistance is a hallmark of T2DM, leading to down-regulation of insulin signaling pathways (at the post-receptor level) in these tissues [139]. Of the diabetic population, 12% are diagnosed with type 1 diabetes, 80% with T2DM, and 8% with pancreatic diabetes (acute and chronic) [140]. About 80% of the PC population have insulin resistance or frank diabetes and are diagnosed at the metastasis stage. However, recent-onset diabetic patients developing diabetes at later age (average age greater than or equal to50) accompanied with weight loss and exceesive exocrine damage (PC associated diabetes mellitus) were higher risk for PC than long term diabetic population [141]. Pharmacological therapies like metformin (lowers blood glucose and insulin levels), sulfonylurea (promotes secretion of insulin from the pancreas) and insulin analogues (glargine) are available to treat diabetes [142, 143]; however these treatments often fail after prolonged usage. However, a case-control study at M.D. Anderson Cancer Center from 2004 to 2008 recruited 973 PDAC patients among them 259 were diabetic. The diabetic patients who received metformin had a lower risk of PC compared to those who were not given metformin; whereas, insulin or insulin secretagogues administered diabetic patients had a higher risk of PC [144].

As mentioned, T2DM is also a major risk factor for several cancers including PC. Epidemiological studies indicate that T2DM patients have a 1.8-fold increased risk for PC development [145]. However, the literature suggests that insulin resistance and diabetes may be a consequence of PC (up to 50-80% of cases). Clinical studies reveal that 0.85% (8 out of 2122) to 7% (6 out of 86) of diabetic patients were first diagnosed with PC [146, 147]. In PC, the increase in circulatory FFAs secreted from AT causes lipotoxicity in β-cells, resulting in PC-associated diabetes mellitus (PCDM) [145, 148, 149]. After tumor resection, the increased survival of PC patients was associated with greater insulin sensitivity [150]. Recently, the American Diabetic Association classified PCDM, which is induced by chronic pancreatitis and pancreatic surgery, as type3c diabetes mellitus [151]. Still, evidence describing how diabetes leads to PC or *vice versa* is lacking. Some of the key molecules secreted from AT are being considered for the treatment of PCDM, which we focus on below.

#### Adrenomedullin and extracellular vesicles (exosomes)

Adrenomedullin (AM) is expressed by F-cells of the pancreas and plays a role in PC along with its receptor (adrenomedullin receptor ADMR). In 1993, AM was initially isolated from an adrenal medulla tumor called a pheochromocytoma. It is also expressed in AT and acts on pancreatic β-cells to inhibit insulin secretion; however, its effects on β-cells are poorly understood [152]. The circulatory levels of AM are very low under normal conditions; however, its levels are elevated in PC to cause insulin resistance [153]. Pancreatic beta, endothelial and stellate cells express ADMR. Its autocrine function in modulating tumor growth and progression has been evaluated in certain PC cell lines, i.e. Panc-1, BxPC3, and MPanc96 as well as human PSC and endothelial cells [154]. In a study, treatment with AM antagonist reduced PC tumor growth which indicating that AM plays a role in promoting PC progression. Furthermore, silencing of ADMR inhibited tumor growth and metastasis in liver and lung tissues of xenograft mice [155]. In PCDM, plasma levels of AM were significantly higher compared to diabetic patients alone, and its expression is higher in tumor and hypoxia conditions [152, 156].

AM is transported in the pancreas by extracellular vesicles, which contain proteins, lipids, and nucleic acids and are secreted by all cell types into circulation [157]. These vesicles play an important role in the transportation of biological components to other cells and tissues [158]. Extracellular vesicles form exosomes (30-100 nm) by inward or reverse budding of vesicular bodies called microvesicles (100-1000 nm) or by outward blebbing of membrane and apoptotic bodies (500-2000 nm) [159, 160]. Exosomes derived from PC cells have the capacity to promote metastasis in a tissue such as liver by residing in a pre-metastatic niche. The niche contains macrophage migration inhibitory factor engulfed by Kupffer cells, which induces secretion of fibronectin in the liver. The secreted fibronectin inhibits infiltration of macrophages and neutrophils derived from the bone marrow and promotes tumor growth [161]. PC exosomes control the specific site of organ metastasis by producing integrins, molecules that mediate cell adhesion. For example, Kupffer, lung fibroblast and epithelial cells recognize integrins such as αvβ5, α6β1 and α6β4, respectively, and subsequently recruit PC cells to these organs [162]. PC exosomes can also transfer AM to pancreatic β-cells (via caveolin-dependent endocytosis and micropinocytosis), which causes insulin resistance through ADMR-AM interactions. Furthermore, the presence of exosomal AM results in β-cell damage by increasing the production of reactive oxygen/nitrogen species and by increasing endoplasmic reticular stress markers (Bip and Chop) [163]. PC cell exosomes containing AM enter AT by the same mechanism that occurs in pancreatic β-cells. Internalization of AM results in lipolysis by activation of hormone-sensitive lipase through p38 and MAPK/ERK pathways; the resulting effect is growth and differentiation of the cancer cells [164]. Another similar peptide to AM is AM-2, which was identified in rats in 2004. AM2 has a similar function as AM in promoting angiogenesis, tumor development, progression and metastasis through MAPK signaling [165]. However, no studies have examined the role of AM-2 in PCDM.

#### Vanin and matrix metalloproteinase

Another important molecule is vanin 1 (VNN1, pantetheinase) present on the surface of epithelial and myeloid cells and highly expressed in the gut and liver tissue [166, 167]. VNN1 is mainly responsible for the breakdown of pantetheine to pantothenic acid (vitamin B_5_) and cysteamine [168]. It is actively involved in inflammation, migration, stress, and glucose and lipid metabolism. Alteration of glucose and lipid metabolic pathways in the liver leads to development of insulin resistance and eventual T2DM. Mice exhibiting diet-induced obesity and Zucker diabetic fatty rats (model for T2DM) have more VNN1 activity in plasma as well as higher expression in the liver compared to normal controls [169]. Based on gene expression profiling, PCDM patients express higher VNN1 and MMP9 levels in peripheral blood as compared to patients with T2DM alone [170]. VNN1 reduces inflammation in PCDM by altering the levels of cysteamine and glutathione. VNN1 along with cysteamine protect the pancreatic β-cells from the oxidative stress generated during streptozotocin-induced diabetes in animals [171]. Enhanced γ-glutamylcysteine synthetase activity observed in vanin-1^-/-^ deficient mice with low levels of cysteamine resulted in an accumulation of endogenous glutathione (GSH) levels [172, 173]. By contrast, over expression of VNN1 decreased expression of GSH and PPARγ, resulting in increased oxidative stress in PCDM [174], through an unknown mechanism. These findings suggest that decreased GSH and PPARγ might contribute to islet dysfunction in PCDM and that vanin-1 and MMP9 could serve as novel pharmacological targets to treat early asymptomatic PCDM patients.

### Impact of obesity and diabetes on acinar, ductal and islet cells

The pancreas islets, acinar cells, and ducts of the gland make up approximately 2-3%, 85% and 5% of the volume, respectively. Similar to other organs, pancreas size is regulated by genetic as well as environmental factors (food intake) [175]. Feeding chronic high fat diet to Zucker diabetic fatty rats (model for both obesity and T2DM) showed excessive fat accumulation in pancreatic acinar cells and later resulted in acinar cell injury and pancreatic fibrosis [176]. In another study, feeding high fat or high calorie-diets to *Pdx-1*^Cre^ and LSL-*Kras*^G12D^ mice caused increased PSC activation, stromal fibrosis and infiltration by inflammatory cells [177]. In case of T2DM, both islets and peri-islet exocrine tissue of pancreas have an activated PSC. The activated, as well as quiescent PSC express receptors for insulin and insulin-like growth factor, however in activated PSC; insulin enhances cell proliferation and production of extracellular matrix proteins as compared to quiescent PSC [178]. Moreover, obese and T2DM patients show a ten-folds and four-folds increase in pancreatic ductal cell replication (more Ki67 expression), respectively. The increased pancreatic ductal cell replication is a risk factor towards pancreatitis and pancreatic cancer in obesity and or type 2 diabetes subjects [179].

### Obesity and diabetes associated PC stem cells

Studies are suggesting that tumor initiation, progression, and resistance to chemotherapy is due to the presence of a small subset of a cell population within the tumor called cancer stem cells [180–182]. The presence of stem cell markers in normal pancreas might be involved in the progression of PC and resistance to drugs [183]. In obesity, leptin treatment affected PC progression and increased pancreatic cancer stem cell markers such as CD24/CD44/ESA, ALDH, CD133, and Oct-4. Further, the expression of leptin receptor was decreased by tumor suppressor micro RNAs that specifically target pancreatic stem cell markers (Met, ABCB1, and CD44) to reduce their expression [184]. Leptin is also involved in the growth of PC tumorspheres and resistance to the chemotherapeutic drug (gemcitabine) [185] by increasing the stem cell markers (CD24, CD44, ESA, CD133, and ALDH) in MiaPaCa-2 PC cell line. Additionally, leptin up-regulates the expression of ABCB1 (an ATP binding transporter protein) in PC tumorspheres suggesting its role in stem cell stimulation and chemoresistance [70]. In case of diabetic population, hyperglycemia is a hallmark of T2DM which stimulates PC by promoting a epithelial to mesenchymal transition and expression of pluripotency stem cell markers (Sox2, Oct4, and Nanog) via activating transforming growth factor-beta 1 [186]. Further, studies are needed to understand the exact molecular mechanisms involved in metabolic diseases associated with PC stem cells.

## Conclusions

Several studies suggest that obesity and T2DM increase the risk for PC development and its pathogenesis. However, mechanistic interplay responsible for development and progression of pancreatic tumor remains obscure. Recent studies on key players associated with obesity and diabetes such as adipocytokines, gut microbiota, adrenomedullin, hyaluronan, vanin and matrix metalloproteinase have deciphered unknown linkage present across PC as well as PCDM. These mediators play central role in promoting obesity-and diabetes-associated pancreatic cancer, however, to date studies involving therapeutic targeting and harnessing their biomarker potential are still in infancy. Henceforth, based on literature survey, we suggest that there is an urgent need to delineate biomarkers as well as therapeutic target(s) involved in the obesity and T2DM associated PC development making inroads to prevent this highly lethal malignancy.

## Abbreviations

AdipoQ: Adiponectin
ADMR: Adrenomedullin receptor
AG: Nab-paclitaxel/gemcitabine
AM: Adrenomedullin
AMPK: Adenosine monophosphate-activated protein kinase
AT: Adipose tissue
ECM: Extracellular matrix
EGFR: Epidermal growth factor receptor
FFA: Free fatty acids
GF: Germ-free
HA: Hyaluronan
HAS: Hyaluronan synthase
HGF: Hepatocyte growth factor
HIF: Hypoxia inducible factor
HRE: Hormone response element
IFP: Interstitial fluid pressure
IL: Interleukin
KC: *Kras*^G12D/+^;*Ptfla*^Cre^
KPC: *Kras* ^*G12D/+*^ *Trp53* ^*R172H/+*^
KRAS: Kirsten rat sarcoma viral oncogene homolog
OBR: Leptin receptor
LPS: Lipopolysaccharide
MMPs: Matrix metalloproteinases
PAG: PEGPH20 + nab-paclitaxel/gemcitabine
PanIn: Pancreatic intra-epithelial neoplasia lesions
PC: Pancreatic cancer
PCDM: PC-associated diabetes mellitus
PDAC: Pancreatic ductal adenocarcinoma
PEGPH20: Pegylated recombinant human hyaluronidase
PFS: Progression-free survival
PSC: Pancreatic stellate cells
T2DM: Type 2 diabetes mellitus
TE: Thromboembolic event rate
TG: Triglycerides
TLR: Toll like receptor
TNF: Tumor necrotic factor
VNN: Vannin
WT: Wild-type
